# Comparative efficacy and cost–utility of combined cataract and minimally invasive glaucoma surgery in primary open-angle glaucoma

**DOI:** 10.1007/s10792-020-01314-7

**Published:** 2020-03-17

**Authors:** Jose Bartelt-Hofer, Steffen Flessa

**Affiliations:** University of Greifswald, Greifswald, Germany

**Keywords:** Primary open-angle glaucoma, POAG, Minimally invasive glaucoma surgery, MIGS, Cost–utility, Cost-effectiveness

## Abstract

**Purpose:**

To assess the comparative efficacy and the long-term cost–utility of alternative minimally invasive glaucoma surgeries (MIGSs) when combined with cataract surgery in patients with primary open-angle glaucoma (POAG).

**Methods:**

Treatment effects, as measured by the 1-year reduction in intraocular pressure (IOP), were estimated with an adjusted indirect treatment comparison. Evidence from randomized clinical trials was identified for four different MIGS methods. A disease-transition model was developed by capturing clinically relevant POAG stages and the expected natural disease evolution. Outcomes of the disease-transition model were the comparative utility [quality-adjusted life years (QALYs)], cost and cost–utility of included strategies in a lifetime horizon.

**Results:**

Estimated 1-year IOP reductions were: cataract surgery − 2.05 mmHg (95% CI − 3.38; − 0.72), one trabecular micro-bypass stent − 3.15 mmHg (95% CI − 5.66; − 0.64), two trabecular micro-bypass stents − 4.85 mmHg (95% CI − 7.71; − 1.99) and intracanalicular scaffold − 2.25 mmHg (95% CI − 4.87; 0.37). Discounted outcomes from the disease-transition model appraised the strategy of two trabecular micro-bypass stents with cataract surgery in the moderate POAG stage as the one providing the greatest added value, with 10,955€ per additional QALY. Improved outcomes were seen when assessing MIGS in the moderate POAG stage.

**Conclusions:**

When indirectly comparing alternative MIGS methods combined with cataract surgery, the option of two trabecular micro-bypass stents showed both a superior efficacy and long-term cost–utility from a German perspective. Moreover, outcomes of the disease-transition model suggest POAG patients to beneficiate the most from an earlier intervention in the moderate stage contrary to waiting until an advanced disease is present.

## Introduction

Glaucoma is a highly prevalent disease in most aging societies [1]. In Germany, the disease affects around 1.44% of the total population [2]; some 10% of severe visual impairments in the country are due to glaucoma [3]. The incidence of blindness related to glaucoma is 2.4 per 100,000 habitants in the nation, which translates into nearly 2000 new cases of blindness per year [4]. These figures are quite representative for European countries and other nations in the fourth or fifth phase of demographic transition [5, 6].

Primary open-angle glaucoma (POAG) is by far the most frequent of all glaucoma types, accounting for about three-quarters of documented cases [6]. Patients with POAG face a degenerative progression of the disease from early to advanced stages; the clinical consequences for patients and the treatment-related costs present a similarly unfavorable evolution. A 5-year cross-sectional study of 137 patients with POAG in 13 ophthalmology clinics in Germany (CoGIS study) suggests that the yearly cost of treating advanced-stage POAG patients is almost double that of treating early stage patients [7]. Considering the same German sample of patients, Wolfram and colleagues demonstrated a 32% deterioration in the patients’ health-related quality of life between early disease and advanced disease [8].

Frequently used clinical interventions for glaucoma include topical medications and three different types of surgery: laser, incisional and minimally invasive. These interventions have been clinically proven to mitigate the disease progression by decreasing intraocular pressure (IOP) [9]. A common treatment pattern following guideline practices begins with the use of topical medications and uses invasive incisional surgery only as a last resort [10]. A step-wise treatment strategy depending on the disease severity has moreover been suggested to optimize cost-effectiveness [11]. Compared with standard POAG incisional surgery, minimally invasive glaucoma surgery (MIGS) is less intrusive, reduces topical medication dependency and has an improved safety profile [9].

For POAG patients with significant cataract, combined glaucoma and cataract surgeries can provide an additive effect in reducing intraocular pressure (IOP) [10]. Cataract surgery likewise promotes a more rapid and enhanced visual recovery, but its use as a stand-alone procedure has nevertheless shown only modest efficacy [10]. Randomized control trials (RCTs) comparing the combined effects of MIGS and cataract surgery allow for indirect comparison of different MIGS alternatives, using cataract surgery as the control arm commonly employed [9].

The use of mathematical, disease-transition models helps to overcome RCT limitations, notably the insufficient patient follow-up, lack of relevant comparative arms and the estimation of costs and relevant clinical outcomes for the patients in the long term. By incorporating all relevant disease stages, such models provide a close estimation of the expected clinical reality of patients. A 2019 review of disease-transition models for POAG revealed a limited number of papers exploring the long-term outcomes of MIGS [12]. The need of patients, physicians and payers to have increased certainty regarding the long-term use of MIGS is crucial for well-informed decision-making.

## Methods

This analysis aims to identify the most effective method of MIGS (measured as the patient’s utility), in consideration of its additional cost. A secondary objective of the study is to estimate the comparative efficacy of MIGS in terms of IOP reduction from baseline to year one, achieved by indirectly comparing RCT data using established statistical methods.

A disease-transition model was the groundwork of the study, built to estimate and compare the long-term utility, costs and cost–utility of alternative MIGS with cataract surgery, or cataract surgery as a stand-alone surgery in patients with POAG. The decision-analytic model incorporated the following data in a single framework: efficacy; natural evolution of the disease; patient-reported outcomes; and direct medical resource consumption. The use of German-specific data was prioritized when possible.

### Disease-transition model

**Fig. 1:**
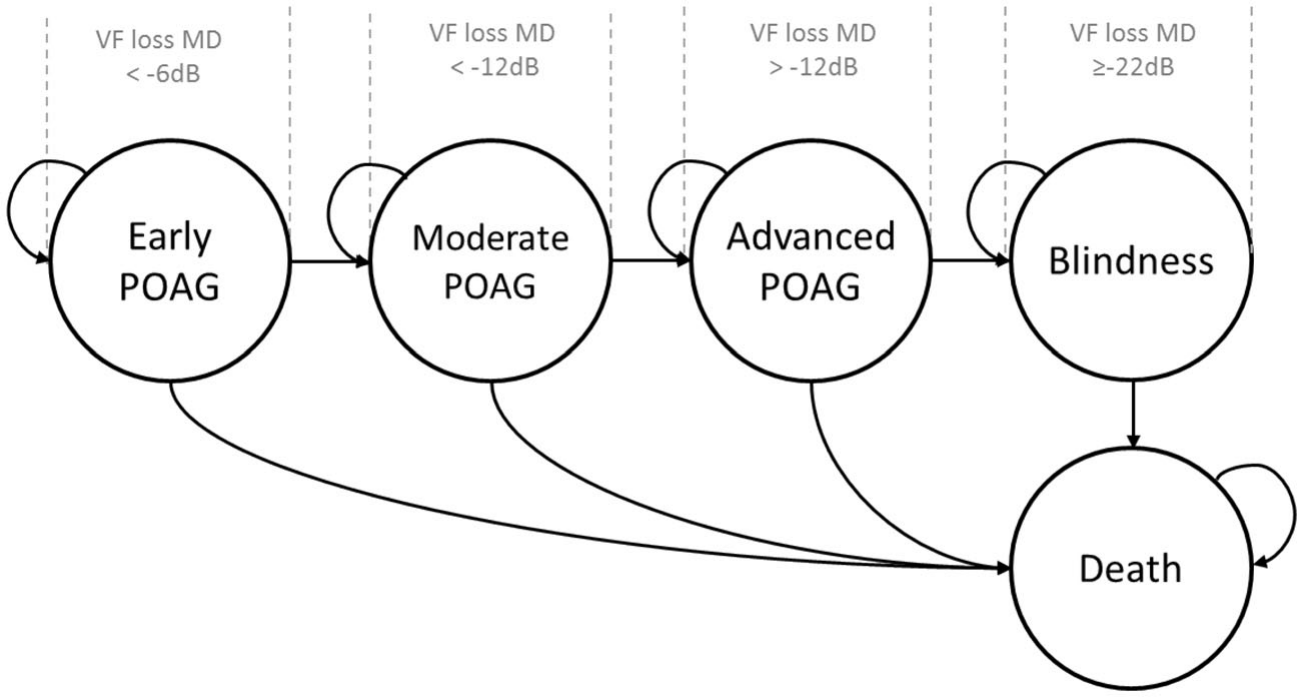
Disease-transition model (Markov) scheme

Clinical stages in the model followed the widely used Hodapp–Parrish–Anderson criteria for classifying the disease according to its severity [13]. POAG stages were therefore divided by assessing the mean deviation (MD) in the visual field (VF) in terms of decibels (dB) between early (< − 6 dB) disease, moderate (< − 12 dB) disease and advanced (> − 12 dB) disease. The stages of death and blindness were additionally incorporated into the model; Heijl et al. [14] defined blindness in POAG as a deviation ≥ − 22 dB in the visual field. The analytical method for the model followed a Markov approach, which is able to capture the transition of patients between mutually exclusive clinical stages during discrete periods of time [15]. The graphical representation of the model is displayed in Fig. 1; the arrows show the possible transition of patients.

Efficacy data were retrieved from RCTs studying the combined effect of MIGS and cataract surgery with at least one year of patient follow-up. A 2017 systematic literature review by Lavia and collaborators identified four alternative MIGS methods: (a) one trabecular micro-bypass stent (TMBS), (b) two TMBSs, (c) suprachoroidal microstent (SMS) and (d) intracanalicular scaffold (IS), with its main characteristics outlined in Table 1 [9].

**Table 1:** List of identified studies (MIGS on top of cataract surgery in RCTs)

MIGS	Study evidence	Reported IOP measures			
		Intervention Mean mmHG (SD)		Control Mean mmHG (SD)	
		Baseline	Year 1	Baseline	Year 1
One trabecular micro-bypass stent	Craven et al. [16]	18.6 (3.4)	17 (2.8)	17.9 (3)	17. (3.1)
	Fea et al. [17]	17.8 (2.7)	14.7 (1.3)	16.7 (3)	15.6 (1.1)
Two trabecular micro-bypass stents	Fernández-Barrientos et al. [18]	24.2 (1.6)	17.6 (2.8)	23.6 (1.5)	19.8 (2.3)
Intracanalicular scaffold	Pfeiffer et al. [19]	18.9 (3.3)	16.1 (3)	18.6 (3.8)	16 (2.8)

The SMS alternative was removed from the analysis after being withdrawn from the market by the manufacturer due to outcomes suggesting higher rates of endothelial cell loss [16]. Two additional hypothetical comparisons were added in order to estimate the effect of treatment classes: (a) one or two TMBSs and (b) combined MIGS.

### Indirect treatment comparison and combined treatment effects

**Fig. 2:**
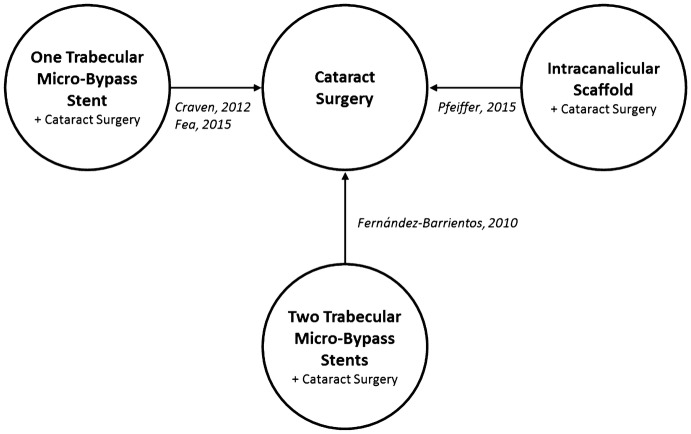
Combined MIGS—RCTs with a common cataract surgery control arm

An adjusted indirect treatment comparison (ITC) was used to overcome missing head-to-head comparative arms in the identified RCTs. The common control arm of cataract surgery as a single procedure allowed for indirect comparison of alternative MIGS. The widely used ITC method described by Bucher and collaborators was used, which allows for an adjusted estimation of the effects without compromising the original trial randomization [17]. In alignment with the evidence found, a total of three ITCs were feasible, as outlined in Fig. 2.

The 1-year change from baseline in IOP (mmHg) was the single homogeneous efficacy endpoint identified for the ITC. When more than one RCT informed the efficacy of an alternative, a single combined treatment effect was estimated using a meta-analysis. In the ITC methods, potential heterogeneity between clinical trials was accounted for. Table 2 gives the final ITC estimates, with additional information on the number of studies informing the effect, and the observed heterogeneity of them (*I*^2^ statistic).

**Table 2:** Indirect treatment comparison and meta-analysis outcomes—1-year mean change from baseline in IOP

Strategy	IOP change (mmHg)			No. RCTs	*I*^2^
	Mean	95% CI low	95% CI upper		
Cataract surgery alone	− 2.05	− 3.38	− 0.72	4	81%
One TMBS with cataract surgery	− 3.15	− 5.66	− 0.64	2	39%
Two TMBSs with cataract surgery	− 4.85	− 7.71	− 1.99	1	NA
IS + cataract surgery	− 2.25	− 4.87	0.37	1	NA
*Exploratory comparisons using meta-analysis effects*					
One or two TMBSs with cataract surgery	− 3.87	− 6.66	− 1.08	3	97%
MIGS with cataract surgery	− 3.49	− 5.52	− 1.46	4	97%

TMBS trabecular micro-bypass stent; IS intracanalicular scaffold; MIGS minimally invasive glaucoma surgeries; mmHg millimeter of mercury; *I*^2^ statistic describing the presence of heterogeneity between studies when a meta-analysis was performed; NA not applicable

### Model inputs and data processing

Model inputs characterize the utility of patients, the efficacy of competing alternatives and the consumption of direct medical resources related to treatment interventions and usual management. Utility scores utilized in the model are German-specific and were sourced from a 2013 study done by Wolfram et al. [8], who employed the health utilities index 3 elicitation instrument [18]. The utility score for blindness was estimated with a least squares regression from all three POAG stages following its decreasing trend. Final values used in the model are presented in Table 3. Utility scores range from 1, a state of perfect health, to 0, the worst possible health scenario.

**Table 3:** Utility values used in the model

Variable	Utility score (SD)	Source
Early POAG	0.85 (0.15)	Wolfram et al. [8]
Moderate POAG	0.75 (0.23)	Wolfram et al. [8]
Advanced POAG	0.58 (0.32)	Wolfram et al. [8]
Blindness	0.46 (0.4)	Least squares estimation

POAG patients in Germany follow a natural evolution of the disease from early to advanced stages, despite medical efforts to mitigate the progression. The probabilities of this natural progression were estimated with the use of German data from the 2013 CoGIS study [7]. The reported 5-year percentages of patients progressing from one stage to another were transformed into yearly transition probabilities, following standard methodology [15]. Final estimated yearly transition probabilities are given in Table 4.

**Table 4:** Yearly transition probabilities for standard of care in Germany

Stage	Probability of staying in current stage (%)	Probability of progressing (%)
Early POAG	91.5	8.5
Moderate POAG	87.6	12.4
Advanced POAG	81.4	18.6

The efficacy of alternative MIGS was evaluated as its capacity to slow the disease progression, as measured by the VF, ultimately increasing the chances of patients staying longer in their current POAG stage. Following the estimations done by Madeiros FA and collaborators, each unit of IOP reduction was associated with an improvement of 0.31% per year in the VF [19]. Comparative VF progression curves for each comparative arm were drawn and later transformed into yearly transition probabilities, using custom mathematical methods [15]. The clinical intervention of interest (i.e., MIGS) was tested in both the moderate and advanced POAG stages, in order to provide further insight into optimal strategies. The natural probability of death in Germany was retrieved from the latest reported mortality table (2017) from the German Statistical Office [20]. In the model, this later probability evolves with the aging of the cohort.

The use of direct medical resources in the model was divided into stage-independent and intervention-related. Stage-independent costs included the conventional use of topical medications, ocular examinations and visits to the ophthalmologist. The frequency and type of resource consumption by POAG stage are German-specific, retrieved from the CoGIS study [7]. Stage-independent costs, presented in Table 5, were therefore estimated as the arithmetic multiplication of the frequency by its associated 2019 cost.

**Table 5:** Stage-independent costs (€)

Disease stage	Early POAG		Moderate POAG		Advanced POAG		Source
	Mean	SD	Mean	SD	Mean	SD	
Medication(s)^a^	230.22	145.40	266.58	145.40	411.98	230.22	Lorenz et al. [7] and Rote Liste [25]
Ocular examinations^b^	34.54	21.23	32.74	16.91	34.90	22.30	Lorenz et al. [7] and Kassenärztliche Bundesvereinigung [26]
Visits to ophthalmologists^c^	58.43	29.21	56.81	21.10	66.54	38.95	
Total	323.19	195.84	356.12	183.41	513.42	291.48	

^a^The average price of eight different combined therapies was estimated (multiplied by its frequency of utilization)

^b^51050—Ophthalmological services (includes Visus, IOP, visual field and cup/disk ratio) (multiplied by its frequency of utilization)

^c^ 06212—Basic ophthalmological flat-rates (60 years)( multiplied by its frequency of utilization)

Table 6 gives the intervention-specific costs, which include the cost of the MIGS surgical procedure and the cataract surgery [21]. Surgical procedure costs include the costs associated with postoperative monitoring and treatment. The surgical cost of MIGS, represented in the study as “intraocular intervention of category V3 (reduction of intraocular pressure by filtering operations: filtration operation: with suture fixated implant, with drain under the conjunctiva),” was sourced from the National Association of Statutory Health Insurance Physicians (*Kassenärztliche Bundesvereinigung*) as considered representative of the operation [21]. In the practice, a co-payment reimbursed by patients following the specific choice of device might apply.

**Table 6:** Intervention-specific costs

Intervention	MIGS surgical procedure (€)^a^	Cataract surgery (€)^b^	Total intervention cost (€)
Cataract surgery alone	0	224.84	224.84
One MIGS device with cataract surgery	413.58	224.84	638.42
Two MIGS devices with cataract surgery	712.5	224.84	937.34

^a^Kassenärztliche Bundesvereinigung 2019 includes the cost of surgery (procedure 31333), and the costs of postoperative monitoring (procedure 31504) and treatment (average of procedures 31718 and 31719)

^b^Kassenärztliche Bundesvereinigung 2019 includes the average price of cataract surgery (procedure 36350), the postoperative monitoring (procedure 36502) and the cost of the personalized anesthesia (procedure 36840)

### Disease-transition model settings

Patients were assumed to begin in the early POAG stage, with an average age of 63 years and comprising 44.2% female patients, as estimated in the CoGIS study [7]. Given the starting age of patients and the life expectancy in Germany, the model adopted a lifetime horizon; transitions between clinical stages were estimated in yearly cycles [22]. Health benefits and costs occurring in the future were discounted at 3% per year after year one, following German guidelines; this notion accounts for temporality, since outcomes occurring in the present are more valuable than those occurring in the future [23]. Ultimate effectiveness of comparative alternatives was measured as quality-adjusted life years (QALYs), a measure that weights the patient’s life years by quality of life. All costs in the analysis were expressed in 2019 euros (€).

Finally, the incremental cost-effectiveness ratio (ICER) was estimated as the quotient of the difference in costs by the difference in effectiveness between the alternative (MIGS + cataract surgery) and the standard therapy (cataract surgery alone), following the formula:

$${\text{ICER}} = \frac{{{\text{Cost alternative}} - {\text{Cost standard therapy}}}}{{{\text{Effectiveness alternative}} - {\text{Effectiveness standard therapy}}}}.$$

This measure has proven to be helpful in interpreting results, as it expresses the incremental cost per unit of QALY gained; in a wide range of alternatives showing better effectiveness but also higher costs against standard of care, the one with the lowest ICER can be depicted as the one with the greatest added value.

### Uncertainty and variability

A number of sensitivity analyses were performed to test the robustness of outcomes, given the potential variability of model inputs. Both deterministic and probabilistic analyses were performed. For the deterministic analysis, the potential impact on the final outcomes of a ± 20% change in key individual inputs of the model was estimated. The probabilistic analysis was conducted in the form of a Monte Carlo simulation; by generating random numbers, this technique draws an alternative set of values per simulation, given the expected distribution of the variable [15].

## Results

**Fig. 3:**
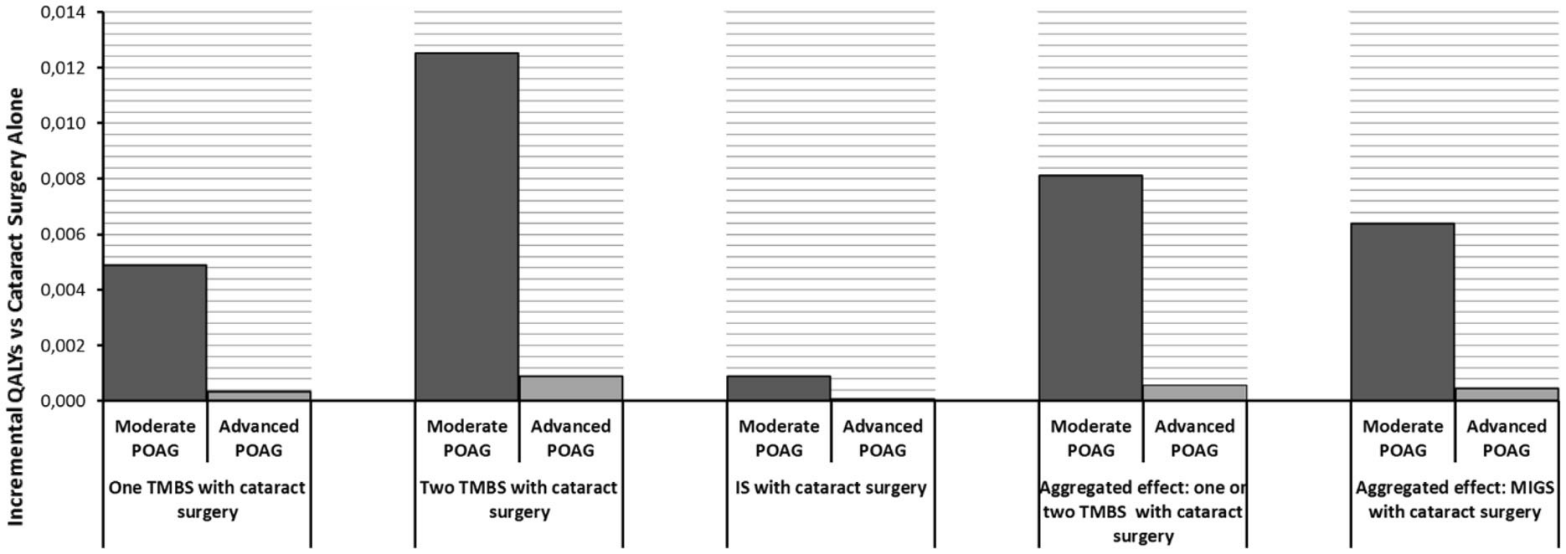
Lifetime incremental effectiveness (QALYs) of MIGS versus cataract surgery alone

**Fig. 4:**
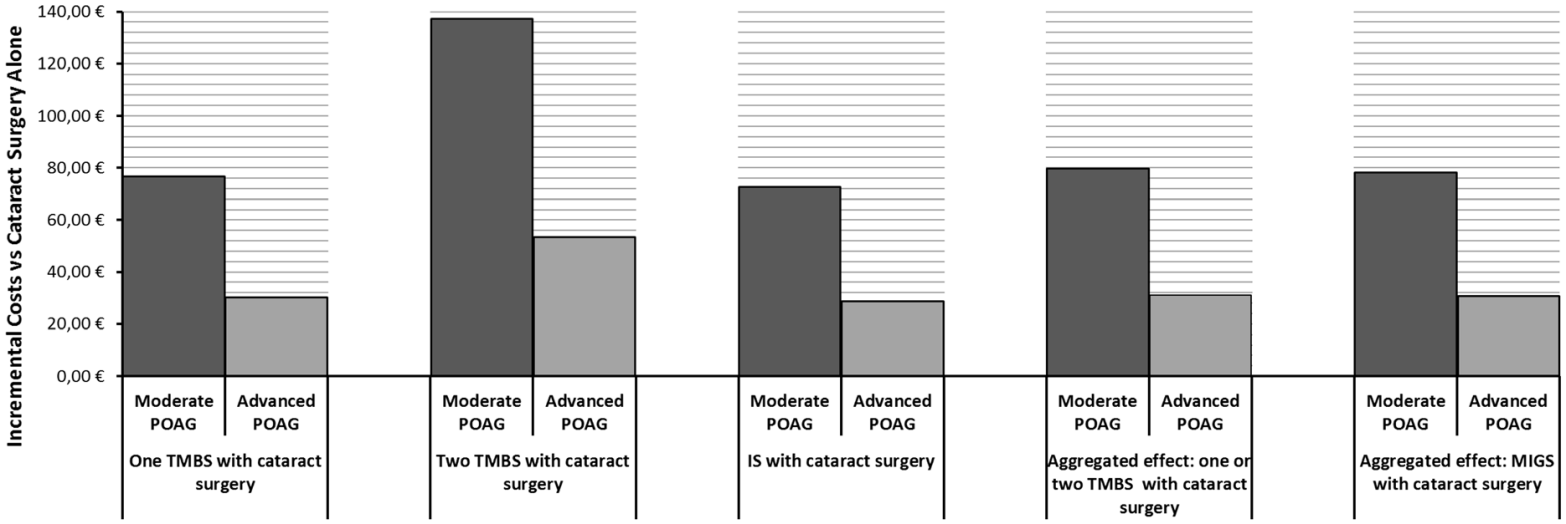
Lifetime incremental costs of MIGS versus cataract surgery alone

Outcomes were estimated based on the alternative scenarios of performing the MIGS in the moderate or in the advanced POAG stages. Figures 3 and 4, respectively, display the incremental effectiveness and incremental costs of the different competing alternatives versus cataract surgery alone. In all cases, the use of MIGS with cataract surgery was related to a gain in both incremental cost and incremental QALY when compared to cataract as stand-alone surgery, with the option of two TMBSs being the most effective but also the most costly. Given the results, it can be affirmed that performing MIGS in the moderate POAG stage is likely associated with improved effectiveness outcomes, but also a higher investment, in comparison with the advanced disease stage.

Table 7 gives the lifetime ICER outcomes of MIGS vs cataract surgery alone when performed either in the moderate or in the advanced POAG stages. A number of conclusions can be drawn from the estimated outcomes. In all cases, performing a MIGS during the advanced POAG stage appeared to minimally improve the utility of patients, resulting in an elevated cost per additional QALY gained. When comparing alternatives in the moderate stage, the option of two TMBSs with cataract surgery displayed both the highest effectiveness and the lowest cost per additional QALY gained, with an estimated ICER of €10,955 per QALY gained.

**Table 7:** Lifetime ICER outcomes of MIGS vs cataract surgery alone in the moderate POAG stage

Strategy	Stage	Costs (€)	Incremental costs (€)	QALYs	Incremental QALYs	ICER
Cataract surgery alone	Moderate POAG	4103.28	0.00	9.72	0.00	–
	Advanced POAG	3917.76	0.00	9.68	0.00	–
One TMBS with cataract surgery	Moderate POAG	4179.83	76.55	9.73	0.00	15,673.41
	Advanced POAG	3947.83	30.07	9.68	0.00	86,942.73
Two TMBSs with cataract surgery	Moderate POAG	4240.53	137.25	9.74	0.01	10,955.20
	Advanced POAG	3971.11	53.36	9.68	0.00	60,269.14
IS with cataract surgery	Moderate POAG	4175.80	72.53	9.72	0.00	82,001.22
	Advanced POAG	3946.51	28.76	9.68	0.00	458,571.31
*Hypothetical comparisons using meta-analysis effects*						
Aggregated effect: one or two TMBSs with cataract surgery	Moderate POAG	4183.06	79.78	9.73	0.01	9840.69
	Advanced POAG	3948.89	31.13	9.68	0.00	54,272.38
Aggregated effect: MIGS with cataract surgery	Moderate POAG	4181.36	78.08	9.73	0.01	12,192.47
	Advanced POAG	3948.33	30.57	9.68	0.00	67,443.84

When seen as a class, MIGS showed consistently better outcomes in the moderate POAG stage at a reasonable additional investment.

### Sensitivity analyses

A number of sensitivity analyses were performed to test the robustness of the outcomes and to identify inputs sensitive to changes. For the deterministic sensitivity analysis, all key inputs of the model were varied by ± 20%. The efficacy of MIGS in delaying the progression from the moderate to advanced POAG stages, and the patient’s experienced utility in the stages of moderate disease, advanced disease and blindness disease were the inputs showing the highest sensitivity in the model. Worth noting is that the cost of the procedures, and notably the cost of MIGS, did not turn out to be as sensitive as expected.

For the probabilistic sensitivity analysis, a Monte Carlo simulation of 10,000 individual iterations was performed. The outcomes of this analysis showed a similar trend to those obtained in the base case; when considering a willingness to pay by payer of 1 GDP per capita in the country (€43,433.87), over 95% of all simulations fall under this threshold, which make MIGS highly cost-effective according to the World Health Organization’s Choosing Interventions that are Cost-Effective project (WHO-CHOICE) [24].

## Discussion

This research assessed the comparative efficacy, and long-term cost, utility and cost–utility of combined MIGS with cataract surgery in patients with POAG, when performed at the either moderate or advanced stages of the disease. The clinical criteria of the present analysis were driven by RCTs, which represent an unbiased source of clinical efficacy of MIGS, but the number of available publications in the literature is narrow. Additional limitations of identified RCTs comprise the meager time horizon of the studies and insufficient inclusion of relevant treatment arms. The long-term utility, cost and cost–utility of alternative MIGS can only be assessed with the use of indirect treatment comparisons and disease modeling techniques that allow for projection of costs and clinical effectiveness, as completed in this study. Under these circumstances, a cost–utility estimation of three different MIGS alternatives and two additional analyses assessing class effects, adopting a German perspective and a lifetime horizon, was feasible.

The treatment of POAG should target to lower the IOP by preserving the patient’s quality of life at treatment costs sustainable for the health system. When evaluated as class in the present study, combined MIGS with cataract surgery proved to increase the IOP drop by 40% while improving the long-term quality of life of patients.

Stage progression in POAG proved to increase the cost of treatment and deteriorate the patient’s quality of life; slowing the evolution of the disease with MIGS will likely reduce this burden. Estimated lifetime costs of competing alternatives proved the addition of MIGS to cataract surgery to almost counterbalance the additional investment in contrast to cataract surgery as stand-alone procedure in the best case.

When facing the challenge to choose a MIGS device, physicians and decision-makers should acknowledge for both robust clinical trials and long-term expected outcomes in terms of cost–utility. The ultimate ratio that combines in a single digit all these factors is the ICER; in light of the present study, two MIGS alternatives demonstrated to be highly cost-effective (under the threshold of 1 German GDP per capita), namely one TMBS and two TMBSs combined with cataract surgery. The option of combined IS and cataract surgery was an option dominated by these above-listed alternatives; one might suggest it should not be the preferred option of MIGS.

Utilizing meta-analysis treatment effects that displayed considerable heterogeneity is a limitation of the present study that could be only mitigated by accounting for it in the choice of statistical methods and by running probabilistic analyses. Moreover, the device selection for a MIGS might vary the individual cost of the procedure. An additional limitation relates to the single homogeneous endpoint for performing indirect treatment comparisons, namely the 1-year change in IOP. This later endpoint had to be translated into a comprehensive input to display how the alternative MIGS slowed the disease progression in real life (i.e., the VF). The need for re-operation or other long-term side effects of MIGS are jet to be proven in the future.

This study adopted a German payer perspective, with the incorporation of costs, patient utilities and natural probabilities of progression, its transferability to other country settings might be limited.

## Conclusion

MIGS represents an innovative approach to handle patients with POAG, with a promising safety and efficacy profile. The progression of the disease is associated with both an increase in the treatment cost of patients and a decrease in their quality of life; therefore, the study of MIGS achieves a twofold benefit in this long-term cost–utility evaluation.

The evidence underlying this analysis suggests that the combined procedures cataract surgery and MIGS performed on moderate POAG could be beneficial in Germany, mitigating the additional cost of the MIGS implant. In contrast, the marginal gain in utility for patients already in the advanced POAG stage may not sufficiently justify the additional investment. When studied in the moderate POAG stage, the option of two TMBSs with cataract surgery proved to be the alternative with the lowest cost per additional QALY gained, making it the preferred choice of treatment based on cost–utility criteria.

A hypothetical comparison of the aggregated class effect of MIGS draws similar conclusions, by encouraging that MIGS be performed in the moderate stage, to maximize cost–utility. Several analyses tested the robustness of this later conclusion by changing key parameters of the model, demonstrating that treatment efficacy and stage-related quality of life play an important role in the outcomes.

## References

[1] Tham YC, Li X, Wong TY, Quigley HA, Aung T, Cheng CY (2014). Global prevalence of glaucoma and projections of glaucoma burden through 2040: a systematic review and meta-analysis. Ophthalmology.

[2] Höhn R, Nickels S, Schuster AK, Wild PS, Münzel T, Lackner KJ, Schmidtmann I, Beutel M, Pfeiffer N (2018). Prevalence of glaucoma in Germany: results from the Gutenberg Health Study. Graefes Arch Clin Exp Ophthalmol.

[3] Deutsche Ophthalologische Gesellschaft (2015) Stellungnahme zur Glaukomfrüherkennung. https://www.dog.org/wp-content/uploads/2015/11/SN-Glaukom-August-2015.pdf. Accessed 28 Sept 2018

[4] Berufsverband der Augenärzte Deutschlands (2019) Glaukom. https://cms.augeninfo.de/nc/hauptmenu/presse/statistiken/statistik-glaukom.html. Accessed 14 May 2019

[5] Day AC, Baio G, Gazzard G, Bunce C, Azuara-Blanco A, Munoz B, Friedman DS, Foster PJ (2012). The prevalence of primary angle closure glaucoma in European derived populations: a systematic review. Br J Ophthalmol.

[6] Kapetanakis VV, Chan MP, Cook DG, Owen CG, Rudnicka AR (2016). Global variations and time trends in the prevalence of primary open angle glaucoma (POAG): a systematic review and meta-analysis. Br J Ophthalmol.

[7] Lorenz K, Wolfram C, Breitscheidel L, Shlaen M, Verboven Y, Pfeiffer N (2013). Direct cost and predictive factors for treatment in patients with ocular hypertension or early, moderate and advanced primary open-angle glaucoma: the CoGIS study in Germany. Graefes Arch Clin Exp Ophthalmol.

[8] Wolfram C, Lorenz K, Breitscheidel L, Verboven Y, Pfeiffer N (2013). Health- and vision-related quality of life in patients with ocular hypertension or primary open-angle glaucoma. Ophthalmologica.

[9] Lavia C, Dallorto L, Maule M, Ceccarelli M, Fea AM (2017). Minimally-invasive glaucoma surgeries (MIGS) for open angle glaucoma: a systematic review and meta-analysis. PLoS ONE.

[10] European Glaucoma Society (2017). European Glaucoma Society Terminology and Guidelines for Glaucoma, 4th Edition—Chapter 3: Treatment principles and options. Br J Ophthalmol.

[11] Paletta Guedes RA, Paletta Guedes VM, de Mello Gomes CE, Chaoubah A (2016). Maximizing cost-effectiveness by adjusting treatment strategy according to glaucoma severity. Medicine.

[12] Bartelt-Hofer J, Ben-Debba L, Flessa S (2019). Systematic review of economic evaluations in primary open-angle glaucoma: decision analytic modeling insights. Pharmacoecon Open.

[13] Remo S, Vessani RM (2009). Staging glaucoma patient: why and how?. Open Ophthalmol J.

[14] Heijl A, Aspberg J, Bengtsson B (2011). The effect of different criteria on the number of patients blind from open-angle glaucoma. BMC Ophthalmol.

[15] Briggs A, Claxton K, Sculpher M (2006). Decision modelling for health economic evaluation.

[16] Craven ER, Katz LJ, Wells JM, Giampocaro JE, iStent Study Group (2012). Cataract surgery with trabecular micro-bypass stent implantation in patients with mild-to-moderate open-angle glaucoma and cataract: two-year follow-up. J Cataract Refract Surg.

[17] Fea A, Zola M, Pignata G, Cannizzo P, Lavia C, Rolle T, Gignlolo F (2015). Micro-bypass implantation for primary open-angle glaucoma combined with phacoemulsification: 4-year follow-up. J Ophthalmol.

[18] Fernández-Barrientos Y, García-Feijoó J, Martínez-de-la-Casa JM, Pablo LE, Fernández-Pérez C, García Sánchez J (2010). Fluorophotometric study of the effect of the glaukos trabecular microbypass stent on aqueous humor dynamics. Invest Ophthalmol Vis Sci.

[19] Pfeiffer N, Garcia-Feijoo J, Martinez-de-la-Casa JM, Larrosa JM, Fea A, Lemij H, Gandolfi S, Schwenn O, Lorenz K, Samuelson TW (2015). A randomized trial of a Schlemm's canal microstent with phacoemulsification for reducing intraocular pressure in open-angle glaucoma. Ophthalmology.

[20] Alcon (2018) CyPass^®^ Micro-stent market withdrawal. https://www.alcon.com/cypass-recall-information. Accessed 07 May 2019

[21] Bucher HC, Guyatt GH, Griffith LE, Walter SD (1997). The results of direct and indirect treatment comparisons in meta-analysis of randomized controlled trials. J Clin Epidemiol.

[22] Horsman J, Furlong W, Feenly D, Torrence G (2003). The Health Utilities Index (HUI): concepts, measurement properties and applications. Health Qual Life Outcomes.

[23] Medeiros FA, Alencar LM, Sample PA, Zangwill LM, Remo S, Weinreb RN (2010). The relationship between intraocular pressure reduction and rates of progressive visual field loss in eyes with optic disc hemorrhage. Ophthalmology.

[24] DESTATIS, Statistisches Bundesamt (2017) Genesis-online Datenbank, Sterbetafeln. https://www-genesis.destatis.de/genesis/online/data;sid=CF3A503867496AA5F9B1C1047137DBE4.GO_1_3?operation=statistikenVerzeichnisNextStep&levelindex=0&levelid=1554470557411&index=1&structurelevel=3. Accessed 05 Apr 2019

[25] Arzneimittelinformationen für Deutschland (2019) Rote Liste. https://www.rote-liste.de/. Accessed 15 Feb 2019

[26] Kassenärztliche Bundesvereinigung (2019) Online-version des Einheitlicher Bewertungsmassstab. https://www.kbv.de/html/online-ebm.php. Accessed 14 Feb 2019

